# Do not Bet on the Unknown Versus Try to Find Out More: Estimation Uncertainty and “Unexpected Uncertainty” Both Modulate Exploration

**DOI:** 10.3389/fnins.2012.00150

**Published:** 2012-10-16

**Authors:** Élise Payzan-LeNestour, Peter Bossaerts

**Affiliations:** ^1^Australian School of Business, University of New South WalesSydney, NSW, Australia; ^2^California Institute of TechnologyPasadena, CA, USA

**Keywords:** estimation uncertainty, unexpected uncertainty, Bayesian learning, exploration bonuses, restless bandit problem

## Abstract

Little is known about how humans solve the exploitation/exploration trade-off. In particular, the evidence for uncertainty-driven exploration is mixed. The current study proposes a novel hypothesis of exploration that helps reconcile prior findings that may seem contradictory at first. According to this hypothesis, uncertainty-driven exploration involves a dilemma between two motives: (i) to speed up learning about the unknown, which may beget novel reward opportunities; (ii) to avoid the unknown because it is potentially dangerous. We provide evidence for our hypothesis using both behavioral and simulated data, and briefly point to recent evidence that the brain differentiates between these two motives.

## 1 Introduction

Learning to choose between multiple unknown prospects, in the hope of eventually exploiting the most rewarding ones, is a difficult yet fundamental problem. It involves a trade-off between two competing courses of action: to exploit known options that are believed to yield the best outcomes versus to explore unknown alternatives that may be even more rewarding.

Little is known about how humans solve this trade-off. In particular, the determinants of exploratory decisions remain underspecified. In the model-free reinforcement learning framework, exploration is undirected, i.e., it boils down to introducing *annealing* in the choice rule, whereby the agent either periodically chooses at random, or increases stochasticity of choice when options have similar estimated values (Sutton and Barto, 1998). A more efficient strategy may consist of directing exploration to those options about which the agent is most uncertain about the expected value (e.g., Gittins and Jones, 1974; Kakade and Dayan, 2002; Huettel et al., 2006; Cohen et al., 2007). Whether individuals implement such uncertainty-driven exploration remains an open question.

The existing evidence for uncertainty-driven exploration is mixed. Recently, (Frank et al., 2009) found that participants in a reward learning task were “*ambiguity seekers*,” i.e., they strategically explored the least well known options, with large individual differences that varied as a function of prefrontal cortex genetic function. In a follow-up imaging study (Badre et al., 2012) revealed the rostrolateral prefrontal cortex (RLPFC) to signal estimation uncertainty only in the participants identified as ambiguity seekers. Furthermore, Cavanagh et al. (2011) showed with EEG that these uncertainty signals are represented prior to the decision, which further suggests they drive ambiguity seeking choice. However, these results may appear at odds with the ample evidence, from Allais (1953) to Payzan-LeNestour and Bossaerts (2011), that individuals direct exploration to the *least* uncertain options, thereby shying away from coping with the unknown (“*ambiguity aversion*”). A neurobiological foundation for ambiguity aversion has recently been laid (see, e.g., Hsu et al., 2005; Huettel et al., 2006; Levy et al., 2010).

The current study attempts to reconcile these findings. As noted by Cavanagh et al. (2011) and Badre et al. (2012), the phenomenon of ambiguity aversion could be parasitic on *sticky choice* – the behavioral pattern consisting in repeating the same choice regardless of reward statistics. The idea is that would the agent preferentially choose the options he repeatedly chose in the past, he may behave this way either because he is ambiguity averse (those repeatedly sampled options are the least uncertain), or merely because he tends to stick to prior choices. A related concern is that unless modeled explicitly, sticky choice makes it hard to identify any positive influence of estimation uncertainty on exploration. However, sticky choice appeared to be a second-order phenomenon in Payzan-LeNestour and Bossaerts’s (2011) task. Besides, the evidence for ambiguity aversion documented in Payzan-LeNestour and Bossaerts (2011) still prevailed after accounting for sticky choice in the behavioral models used in that study, which rules out the possibility that such ambiguity averse behavior merely be “sticky choice in disguise^1^.”

The current study proposes a novel hypothesis about exploration that helps reconcile the findings of Payzan-LeNestour and Bossaerts (2011) and Frank et al. (2009)/Cavanagh et al. (2011)/Badre et al. (2012; henceforth, FCB). According to this hypothesis, uncertainty-driven exploration involves a dilemma between two motives: (i) to speed up learning about the unknown, which may beget novel reward opportunities; (ii) to avoid the unknown because it is potentially dangerous. The first motive is connected with the notion of *curiosity* (van Dijk and Zeelenberg, 2007) whereas the second is connected with cautiousness. Below we will briefly point to recent evidence that the brain differentiates between these two motives. We argue that in the task used in FCB, both motives prevailed, though behavior was only influenced by the first motive, which dominated the second one. The second motive was somewhat muted because the potential monetary losses in that task were relatively small, especially compared to those in the task used in Payzan-LeNestour and Bossaerts (2011), where the payoffs were highly skewed. The two motives were – arguably – equally important in that task. This claim may seem strange at first: that ambiguity aversion prevailed would rather suggest that the second motive dominated, i.e., that the cautionary signal not to bet on things unknown countervailed the directive to sharpen the learning about the unknown. But the current study shows that our subjects were in fact both ambiguity averse and novelty seekers.

We flesh out new explanations of subject behavior in Payzan-LeNestour and Bossaerts’s (2011) task, a *restless* (Wittle, 1988) multi-armed bandit in which reinforcement contingencies jumped at unsignaled times. In this kind of changing environment, the directive to speed up learning is primarily relayed through *unexpected uncertainty* (Yu and Dayan, 2005) signals: when jump likelihood is high (i.e., unexpected uncertainty is great), the motivation to explore to find out novel reward opportunities ought to be maximal. We fitted to subject behavior in the task a new model that allows trial-by-trial estimates of both estimation uncertainty and unexpected uncertainty. This model assumes that the agent, in addition to directing exploration to the options for which estimation uncertainty is minimal, also directs exploration to the options for which unexpected uncertainty is maximal. This model markedly improved the fit of the previously developed ambiguity averse model, which Payzan-LeNestour and Bossaerts (2011) found to be the best fit to behavior in the task. This finding shows that in our experiment, unexpected uncertainty modulated the “curiosity motive” (i), while estimation uncertainty modulated the “cautiousness motive” (ii).

We also show with simulated data that the behavior consisting of mixing ambiguity aversion with novelty seeking is natural viewed from the evolutionary fitness principle. We conducted a number of simulations of behavior in the foregoing restless bandit task, in order to compare economic performance of a variety of models that allowed alternate kinds of uncertainty-driven exploration (specifically, ambiguity seeking, ambiguity aversion, novelty seeking, and a mixture of the latter two). Our simulated data reveal ambiguity aversion to improve economic performance in the task compared to ambiguity seeking. This result questions the standard claim that ambiguity aversion [i.e., motive (ii) in the above dilemma] is irrational. We further found that the behavior that mixes ambiguity aversion with novelty seeking fared best in the task. This suggests that both stated motives (i) and (ii) can be vindicated on the grounds of evolutionary fitness.

## 2 Materials and Methods

### 2.1 Experimental task

The current study builds on the restless bandit task originally described in Payzan-LeNestour and Bossaerts (2011) as well as Payzan-LeNestour (2012), where full task details are provided^2^. In what follows we focus on the task features relevant for the current study.

The task is a six-armed bandit. Three arms are blue and three are red. Color is visible. At each trial, every arm generates one of three possible outcomes: 1, −1, or 0 CHF^3^ for the blue arms; 2, −2, or 0 CHF for the red arms. At each trial, the agent selects one arm and immediately receives the outcome returned by the chosen arm. He is not told the outcomes returned by the other arms.

Our bandit is restless: while absolute expected value is constant for each arm, the sign of expected value occasionally flips, thus arms switch from having positive to negative expectation and back. The flips in the outcome probabilities occur without notice. Specifically, changes are instantiated with two independent Bernoulli processes, one for the blue arms and one for the red. For each process and at each trial, either “jump” or “no jump” occurs. When jump occurs for one of the two colors, then at the three arms of this color, the probabilities of two outcomes flip. Jump frequency is higher for the red arms than for the blue ones (1/4 versus 1/16), whereby unexpected uncertainty is higher for the red arms on average.

The subject knows that outcome probabilities will change without warning during the experiment (he also knows red arms are more unstable but is not told the jump probabilities), which leads him to track unexpected uncertainty throughout the task, as we show elsewhere (Payzan-LeNestour et al., in preparation). The same study reveals subjects to track estimation uncertainty as well. One distinctive characteristic of our design is that the levels of both estimation uncertainty and unexpected uncertainty vary substantially during the task. Unexpected uncertainty levels vary from high, upon jumps, to low, during the stable phases. Also, because learning has to be reset after each jump, estimation uncertainty remains significant throughout the task. This manipulation renders the trial-by-trial estimation of both uncertainty components meaningful. Importantly, participants in our task did estimate these components, contrary to that in prior studies where unexpected uncertainty appeared to be artifactually maximal throughout the task (e.g., Daw et al., 2006; Jepma and Nieuwenhuis, 2011)^4^.

### 2.2 Computational models

The current study augments the Bayesian model described in Payzan-LeNestour and Bossaerts (2011). Here we briefly point to the essentials of that model. The model learns the outcome probabilities of the six arms through a natural sampling scheme (analogous to the one proposed in Hirayama et al. (2004, 2006) and Quinn and Karny (2007) which exponentially discounts (“forgets”) the past outcomes returned by a given arm after discovering the arm has jumped. A key feature of the model is that the discount factor is adjusted on the spot on each trial *T*. It equals the likelihood that no jump occurred at trial *T*, i.e., it quantifies the “confidence in stability” at trial *T*. Since jumps are color-specific in the task, the model uses two discount factors, one for the red arms, λ*_red_*(*T*), and one for the blue, λ*_blue_*(*T*). λ*_red_*(*T*) (resp. λ*_blue_*(*T*)) is thus proportional to the strength of evidence that red arms (resp. blue arms) did not change at trial *T*.

Exponential discounting of the past has the appealing property of being related to *leaky-integration processes*, which have been commonly used to model neuronal dynamics in a changing environment (e.g., Sugrue et al., 2004). So this kind of “forgetting Bayesian” model is both a good descriptive model of behavior (as shown in Payzan-LeNestour and Bossaerts, 2011) and a good model of neuronal dynamics (as argued in Yu and Cohen, 2009)^5^.

For each arm *i* and at each trial *T*, the model computes *Q*(*i,T*), the expected value (i.e., the sum of the three possible outcomes weighted by their estimated probabilities of occurrence). The model thus assumes participants were risk neutral and did not distort the outcome probabilities, which is at odds with a number of theories (e.g., *Prospect Theory*). The motivation for this modeling choice is both parsimony and agnosticism about whether/how individuals actually distort probabilities (which reflects disagreement in the literature^6^).

Action selection in the task is modeled with the *softmax rule*. According to this rule, option *i* is chosen with probability *P_iT_* which is proportional to the exponential of the value of arm *i*:

$$P_{iT}\propto \text{exp}\beta Q_{i,T}.$$

β (the *inverse temperature*) is a free parameter controlling the degree to which the subject makes exploitative choices versus exploratory ones.

Payzan-LeNestour and Bossaerts (2011) report that their behavioral data were best fit with the assumption that subjects tracked the level of estimation uncertainty of the options, in order to strategically explore options with minimal estimation uncertainty on a given trial. Such ambiguity averse behavior is accomplished by subtracting from the *Q*-value entering the softmax rule an exploration “malus” proportional to the level of estimation uncertainty:

$$Q_{iT}\leftarrow Q_{iT}-eu_{iT},$$

where *eu_iT_* is the level of estimation uncertainty about option *i* at trial *T*, quantified in terms of the width (variance or entropy) of the posterior probability distribution tracked by the Bayesian learner (cf. Yoshida and Ishii, 2006; Behrens et al., 2007 and Payzan-LeNestour and Bossaerts, 2011). The width of the distribution reflects the subject’s uncertainty regarding option value. Early in learning, the width is larger (and uncertainty higher) than later is learning.

The alternate “ambiguity seeking” model assumes that subjects guided exploration toward the options for which estimation uncertainty was maximal, whereby they explored the least well known options. This behavior is instantiated by adding to the *Q*-value an exploration bonus proportional to the level of estimation uncertainty:

$$Q_{iT}\leftarrow Q_{iT}+eu_{iT}.$$

The two previous models modulate exploration as a function of estimation uncertainty. We also developed a model featuring a novel kind of uncertainty-driven exploration, to formalize the idea – previously suggested by Cohen et al. (2007) – that exploration ought to be modulated by unexpected uncertainty. Specifically, when reinforcement contingencies change abruptly over time, survival depends on constant adaptation to such changes. This adaptation requires that the agent increases exploration when he deems the environment to be novel (i.e., when unexpected uncertainty is high), in accordance with our stated motive (i) above. We refer to this behavior as “novelty seeking” (to be distinguished from ambiguity seeking as previously defined). In the context of our multi-armed bandit task, the novelty seeking model directs exploration to the arms that have most probably changed. What follows describes how this behavior is accomplished. Without loss of generality, suppose the arm that is tried out at trial *T* is a red one. The model adds to the value of the two red options not currently sampled an exploration bonus proportional to the level of unexpected uncertainty:

$$Q\left(i,T\right)\leftarrow Q\left(i,T\right)+\left(1-\lambda_{red}\left(T\right)\right),$$

where 1 − λ*_red_*(*T*) is the level of unexpected uncertainty about the red options at trial T, quantified in terms of the likelihood that red options did change at trial *T*. To further increase novelty seeking after a jump has been detected, the model also penalizes the value of the arm that is currently tried out, in proportion to the level of unexpected uncertainty at the current trial: *Q*(*i*,*T*) ← *Q*(*i*,*T*) − (1 − λ*_red_*(*T*)).

According to the hypothesis stated in the Introduction, both motives (i) and (ii) influence exploratory decisions. To reflect this, the “hybrid model” combines ambiguity aversion and novelty seeking by modifying the *Q*-value of the two red options not currently sampled as follows:

$$Q\left(i,T\right)\leftarrow Q\left(i,T\right)-eu_{iT}+\left(1-\lambda_{red}\left(T\right)\right),$$

while the value of the arm that is currently tried out is modified as follows: *Q*(*i*,*T*) ← *Q*(*i*,*T*) − *eu_iT_* − (1 − λ*_red_*(*T*)). This hybrid model is the readout of the aforementioned dilemma in the context of the current task: unexpected uncertainty modulates motive (i) while estimation uncertainty modulates motive (ii).

Note that the foregoing models put equal weight on the *Q*-value and uncertainty components. The motivation for this particular modeling choice is parsimony; the relative weights can be changed without changing the essence of the schemes. Specifically, to ensure that our results are robust, for each of the four models above, we tested several alternate models that have a different relative weighting on the *Q*-value component vis-a-vis the uncertainty component(s). These alternative models led to similar results.

### 2.3 Evaluating model fit to behavioral data

We fitted the two new models introduced by the current study (the novelty seeker and hybrid models) to the choice data of Payzan-LeNestour and Bossaerts (2011), using maximum likelihood estimation. Only one parameter (the inverse temperature β) needed to be estimated. We allowed this estimated parameter to vary across participants. We compared the log-likelihoods of each model to the one of the ambiguity averse model (the best fit in Payzan-LeNestour and Bossaerts, 2011) which we use as benchmark here.

### 2.4 Evaluating model fitness in simulated data

We compared the average fitness of the ambiguity averse, ambiguity seeking, novelty seeker, and hybrid models, in a set of 500 simulations of the task, each comprised of 500 trials (the length of our experimental sessions). Here the gage of fitness is the economic performance, i.e., the money accumulated in the 500 trials of the task, averaged across the 500 simulations. For each model, we ran the set of 500 simulations for different values of β, which allowed us to assess the fitness as a function of β.

## 3 Results

### 3.1 Behavioral

**Figure 1:**
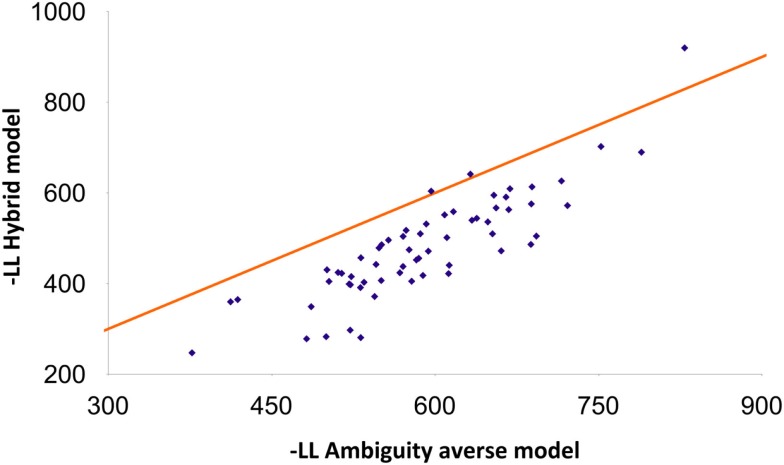
**Comparative fits of the ambiguity averse and hybrid models**. The comparison of the fits is based on the negative log-likelihood (-LL) criterion. Each data point corresponds to one subject (500 samples on average per subject). The hybrid model fits better when the data point is below the 45° line.

The novelty seeker model fitted choices better than the benchmark (ambiguity averse model) in the vast majority (95%) of the participants. A *paired t-test* based on the difference between the negative log-likelihoods of the benchmark and novelty seeker models leads to the conclusion that the novelty seeker model fitted subject behavior better than the benchmark (*p* < 0.001; *N* = 62). For 82% of the participants, the hybrid model fitted subject behavior better than the novelty seeker model. The former significantly outperformed the latter according to a paired *t*-test (*p* < 0.001). Figure 1 reports the negative log-likelihood of the hybrid model, related to that of the benchmark.

### 3.2 Simulations

**Figure 2:**
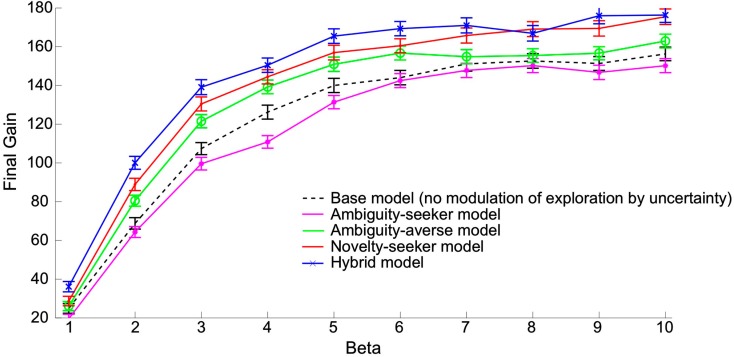
**Economic performances of models featuring different kinds of uncertainty-driven exploration, as a function of the inverse temperature**. Each point reports the economic performance averaged across 500 simulations of 500 trials each. Performance is measured by the amount of money accumulated till the 500th trial (“final gain”). X-axis: β parameter (inverse temperature in the softmax rule). Y-axis: average final gain across 500 simulations. Star (*): performance of the ambiguity seeker model. Circle (o): performance of the ambiguity averse model. Dot (.): performance of the novelty seeker model. Cross (×): performance of the hybrid model. The hybrid model combines ambiguity aversion and novelty seeking as described in the main text. Dashed line: performance of the base model in which there is no uncertainty-driven exploration (for reference). Vertical bars represent standard errors.

Figure 2 shows that in our simulations, the ambiguity averse model performed uniformly better than not only the ambiguity seeking model but also the model that excludes any kind of modulation of exploration by uncertainty (“base model”^7^). The novelty seeker model outperformed the ambiguity averse model, and the hybrid model performed best overall. The standard error of the economic performance is of the same order of magnitude across all models.

## 4 Discussion

Both the behavioral and simulated data reported here support the hypothesis stated in the Introduction. Specifically, the evidence suggests that individuals seek to uncover novel reward opportunities [“curiosity motive” (i)] while they also tend to shy away from the unknown [“cautiousness motive” (ii)], and that this behavior is adaptive, at least in the context of the present task.

Note the ways the task used in the current study is atypical in comparison to previous tasks that were used to study exploration (Daw et al., 2006, FCB). In our task, the dynamic contingencies induced unexpected uncertainty about the value of unexplored options. Unexpected uncertainty and estimation uncertainty did vary significantly throughout the task and participants could estimate them on each trial. This allowed the identification of an unexpected uncertainty bonus together with an estimation uncertainty “malus” in subject exploration. By contrast, in an environment that is unexpected uncertainty free, i.e., when the reinforcement contingencies are stationary (like in the task used in FCB), estimation uncertainty modulates both motives (i) and (ii), and behavior is the readout of the dominating motive [arguably (i) in FCB]. Perhaps cautiousness was muted in FCB because participants knew they would not lose much money by exploring. Additionally, as suggested in Cavanagh et al. (2011), the motivation to learn should be maximal when the agent knows he can potentially suppress ignorance, which is in principle the case when things are stable. In contrast, when things change all the time, motive (i) is probably dampened since the “returns on learning” are low.

Strikingly, the dilemma we describe here has been overlooked in prior work in decision neuroscience and machine learning, on the grounds that exploration should be exclusively driven by the directive to find out more (e.g., Gittins and Jones, 1974; Kakade and Dayan, 2002). Yet, the motive to not bet on the unknown, which is perceived as potentially dangerous, may be equally – if not more – important for survival. Our simulated data point to this possibility: the ambiguity averse model fared better than the ambiguity seeker model in our task. Also, the finding that the ambiguity averse model (let alone the novelty seeker and hybrid models) performed better than the primary model, which excludes any kind of modulation of exploration by uncertainty, should caution the generally accepted view in classical decision theory (Savage, 1954) that uncertainty-driven exploration is irrational. For standard valuation theory, any sensitivity to uncertainty is irrational in that it violates one of the most fundamental principles of rational decision making, namely *the sure thing principle*^8^. Our results contradict this view. We find that in the context of natural sampling, being sensitive to uncertainty appears to be beneficial. This may be the reason why humans display such sensitivity, even if this generates choice inconsistencies in other contexts (e.g., the *Ellsberg Paradox*; Ellsberg, 1961). Humans can afford to be “irrational” as long as this shows up only in ecologically irrelevant contexts (like the gambles underlying the Ellsberg Paradox?), and as long as it is adaptive in ecologically relevant contexts (like our natural sampling task).

That ambiguity aversion may play a positive role, in avoiding danger, has been suggested (albeit implicitly) in Hsu et al. (2005), where amygdala was found to encode ambiguity, presumably through “fear signals.” Also, the current evidence that unexpected uncertainty induces novelty seeking in the action selection rule, together with prior evidence that unexpected uncertainty plays a key role in value updating (e.g., Behrens et al., 2007 and Payzan-LeNestour and Bossaerts, 2011), suggests that unexpected uncertainty plays a dual role, as a modulator of learning as well as of action selection. This implies new challenges and opportunities for neurobiological studies. One can envisage unexpected uncertainty to influence learning through the neuromodulator norepinephrine, while it biases choice through changes in serotonin levels. The former would be consistent with Hasselmo (1999), Yu and Dayan (2005), Rutishauser et al. (2006); the latter would be related to Doya (2008).

## Conflict of Interest Statement

The authors declare that the research was conducted in the absence of any commercial or financial relationships that could be construed as a potential conflict of interest.

## Footnotes

^1^Specifically, the data reported in Payzan-LeNestour and Bossaerts (2011) were fitted by a model allowing for both modulation of exploration by ambiguity (ambiguity-seeking or ambiguity-averse – see Results Section for details on the functional forms) and stickiness in choice (i.e., choice probability is biased towards the latest chosen option, with the biasing factor being a free parameter). The value of the weight on the ambiguity component turned out to be negative for the majority (60 out of 62) of the subjects, which implies ambiguity aversion.

^2^Payzan-LeNestour (2012) is available at http://papers.ssrn.com/sol3/papers.cfm?abstract_id=1628657.

^3^Swiss Francs, the currency used in the original experiment.

^4^In these studies, the analysis suggests that participants presumed changes in the reward contingencies would occur at each trial during the task, perhaps because the task instructions were vague about the nature of the changes in the reward contingencies, and in the absence of knowledge, the “worst-case scenario” (maximal instability) is imagined.

^5^Alternate Bayesian schemes could do as well. For instance, eraspou proposes a “Hierarchical Bayesian” model that is equally good at learning outcome probabilities in the current task, compared to the forgetting Bayesian approach. The probability estimates of the two models are strongly correlated. The forgetting Bayesian model is more tractable and particularly suitable for our purpose in the current analysis.

^6^E.g., Trommershäuser et al. (2008) report that subjects in a movement task represented probabilities in a way that was close to perfect (no distortion whatsoever). By contrast Hertwig et al. (2003) document underweighting of the probability of occurrence of rare events, which is at odds with Prospect Theory which states overweighting.

^7^While the superiority of the ambiguity-averse model over the ambiguity-seeker model appears to be robust to the use of different weighting on the Q-value relative to the uncertainty component in the decision rule, the superiority of the ambiguity-averse model over the base model is not. Specifically, in our simulations, the ambiguity-averse model that puts a minimal weight on the Q-value (i.e., that tends to focus on the uncertainty component exclusively) did not outperform the base model.

^8^According to the sure thing principle, if the agent would take a certain action if he knew that an event *E* obtained, and also if he knew that the negation of *E* obtained, then he should take that action even if he knows nothing about E.

## References

[B1] Allais M. (1953). Le comportement de l’homme rationnel devant le risque: critique des postulats et axiomes de l’ecole americaine. Econometrica 21, 503–546 10.2307/1907921

[B2] Badre D. Doll B. B. Long N. M. Frank M. J. (2012). Rostrolateral prefrontal cortex and individual differences in uncertainty-driven exploration. Neuron 73, 595–607 10.1016/j.neuron.2011.12.025 22325209 PMC3285405

[B3] Behrens T. E. J. Woolrich M. W. Walton M. E. Rushworth M. F. S. (2007). Learning the value of information in an uncertain world. Nat. Neurosci. 10, 1214–1221 10.1038/nn1954 17676057

[B4] Cavanagh J. F. Figueroa C. M. Cohen M. X. Frank M. J. (2011). Frontal theta reflects uncertainty and unexpectedness during exploration and exploitation. Cereb. Cortex. 10.1093/cercor/bhr332 PMC4296208 22120491

[B5] Cohen J. D. McClure S. M. Yu A. J. (2007). Should I stay or should I go? How the human brain manages the trade-off between exploitation and exploration. Philos. Trans. R. Soc. Lond. B Biol. Sci. 362, 933–942 10.1098/rstb.2007.2098 17395573 PMC2430007

[B6] Daw N. D. O’Doherty J. P. Dayan P. Seymour B. Dolan R. J. (2006). Cortical substrates for exploratory decisions in humans. Nature 441, 876–879 10.1038/nature04766 16778890 PMC2635947

[B7] Doya K. (2008). Modulators of decision making. Nat. Neurosci. 11, 410–416 10.1038/nn2077 18368048

[B8] Ellsberg D. (1961). Risk, ambiguity, and the Savage axioms. Q. J. Econ. 75, 643–669 10.2307/1884324

[B9] Frank M. J. Doll B. B. Oas-Terpstra J. Moreno F. (2009). Prefrontal and striatal dopaminergic genes predict individual differences in exploration and exploitation. Nat. Neurosci. 12, 1062–1068 10.1038/nn.2342 19620978 PMC3062477

[B10] Gittins J. Jones D. (1974). Progress in Statistics. Amsterdam: North-Holland

[B11] Hasselmo M. E. (1999). Neuromodulation: acetylcholine and memory consolidation. Trends Cogn. Sci. (Regul. Ed.) 3, 351–359 10.1016/S1364-6613(99)01365-0 10461198

[B12] Hertwig R. Barron G. Weber E. U. Erev I. (2003). Decisions from experience and the effect of rare events in risky choices. Psychol. Sci. 15, 534–539 10.1111/j.0956-7976.2004.00715.x 15270998

[B13] Hirayama J. Yoshimoto J. Ishii S. (2004). Bayesian representation learning in the cortex regulated by acetylcholine. Neural Netw. 17, 1391–1400 10.1016/j.neunet.2004.06.006 15541942

[B14] Hirayama J. Yoshimotoa J. Ishii S. (2006). Balancing plasticity and stability of on-line learning based on hierarchical Bayesian adaptation of forgetting factors. Neurocomputing 69, 1954–1961 10.1016/j.neucom.2005.11.020

[B15] Hsu M. Bhatt M. Adolphs R. Tranel D. Camerer C. F. (2005). Neural systems responding to degrees of uncertainty in human decision-making. Science 310, 1680–1683 10.1126/science.1115327 16339445

[B16] Huettel S. A. Stowe C. J. Gordon E. M. Warner B. T. Platt M. L. (2006). Neural signatures of economic preferences for risk and ambiguity. Neuron 49, 765–775 10.1016/j.neuron.2006.01.024 16504951

[B17] Jepma M. Nieuwenhuis S. (2011). Pupil diameter predicts changes in the exploration-exploitation trade-off: evidence for the adaptive gain theory. J. Cogn. Neurosci. 23, 1587–1596 10.1162/jocn.2010.21548 20666595

[B18] Kakade S. Dayan P. (2002). Dopamine: generalization and bonuses. Neural Netw. 15, 549–559 10.1016/S0893-6080(02)00048-5 12371511

[B19] Levy I. Snell J. Nelson A. J. Rustichini A. Glimcher P. W. (2010). Neural representation of subjective value under risk and ambiguity. J. Neurophysiol. 103, 1036–1047 10.1152/jn.00853.2009 20032238

[B20] Payzan-LeNestour E. (2012). Learning to Choose the Right Investment in an Unstable World: Experimental Evidence Based on the Bandit Problem. Swiss Finance Institute Research Paper No. 10–28. 1–51

[B21] Payzan-LeNestour E. Bossaerts P. (2011). Risk, estimation uncertainty, and unexpected uncertainty: Bayesian learning in unstable settings. PLoS Comput. Biol. 7, e1001048 10.1371/journal.pcbi.1001048 21283774 PMC3024253

[B22] Quinn A. Karny M. (2007). Learning for non-stationary Dirichlet processes. Int. J. Adapt. Control Signal Process. 21, 827–855 10.1002/acs.949

[B23] Rutishauser U. Mamelak A. N. Schuman E. M. (2006). Single-trial learning of novel stimuli by individual neurons of the human hippocampus-amygdala complex. Neuron 49, 805–813 10.1016/j.neuron.2006.02.015 16543129

[B24] Savage L. J. (1954). The Foundations of Statistics. New York: Dover Publications, Inc

[B25] Sugrue L. P. Corrado G. S. Newsome W. T. (2004). Matching behavior and the representation of value in the parietal cortex. Science 304, 1782–1787 10.1126/science.1094765 15205529

[B26] Sutton R. S. Barto A. G. (1998). Reinforcement Learning: An Introduction (Adaptive Computation and Machine Learning). Cambridge: The MIT Press

[B27] Trommershäuser J. Maloney L. T. Landy M. S. (2008). Decision making, movement planning and statistical decision theory. Trends Cogn. Sci. (Regul. Ed.) 12, 291–297 10.1016/j.tics.2008.04.010 18614390 PMC2678412

[B28] van Dijk E. Zeelenberg M. (2007). When curiosity killed regret: avoiding or seeking the unknown in decision-making under uncertainty. J. Exp. Soc. Psychol. 43, 656–662 10.1016/j.jesp.2006.06.004

[B29] Wittle P. (1988). Restless bandits: activity allocation in a changing world. J. Appl. Probab. 25, 287–298 10.2307/3214163

[B30] Yoshida W. Ishii S. (2006). Resolution of uncertainty in prefrontal cortex. Neuron 50, 781–789 10.1016/j.neuron.2006.05.006 16731515

[B31] Yu A. J. Cohen J. D. (2009). “Sequential effects: superstition or rational behavior?” in Advances in Neural Information Processing Systems, Vol. 21, eds Koller D. Schuurmans D. Bengio Y. Bottou L. (Cambridge, MA: MIT Press), 1873–1880 PMC4580342 26412953

[B32] Yu A. J. Dayan P. (2005). Uncertainty, neuromodulation, and attention. Neuron 46, 681–692 10.1016/j.neuron.2005.04.026 15944135

